# Alterations of the extracellular matrix in ovarian cancer studied by Second Harmonic Generation imaging microscopy

**DOI:** 10.1186/1471-2407-10-94

**Published:** 2010-03-11

**Authors:** Oleg Nadiarnykh, Ronald B LaComb, Molly A Brewer, Paul J Campagnola

**Affiliations:** 1 Center for Cell Analysis and Modeling, Department of Cell Biology, University of Connecticut Health Center, Farmington, CT 06030, USA; 2 Neag Cancer Center, Division of Gynecologic Oncology, University of Connecticut Health Center, Farmington, CT 06030, USA

## Abstract

**Background:**

Remodeling of the extracellular matrix (ECM) has been implicated in ovarian cancer, and we hypothesize that these alterations may provide a better optical marker of early disease than currently available imaging/screening methods and that understanding their physical manifestations will provide insight into invasion.

**Methods:**

For this investigation we use Second Harmonic Generation (SHG) imaging microcopy to study changes in the structure of the ovarian ECM in human normal and malignant ex vivo biopsies. This method directly visualizes the type I collagen in the ECM and provides quantitative metrics of the fibrillar assembly. To quantify these changes in collagen morphology we utilized an integrated approach combining 3D SHG imaging measurements and bulk optical parameter measurements in conjunction with Monte Carlo simulations of the experimental data to extract tissue structural properties.

**Results:**

We find the SHG emission attributes (directionality and relative intensity) and bulk optical parameters, both of which are related to the tissue structure, are significantly different in the tumors in a manner that is consistent with the change in collagen assembly. The normal and malignant tissues have highly different collagen fiber assemblies, where collectively, our findings show that the malignant ovaries are characterized by lower cell density, denser collagen, as well as higher regularity at both the fibril and fiber levels. This further suggests that the assembly in cancer may be comprised of newly synthesized collagen as opposed to modification of existing collagen.

**Conclusions:**

Due to the large structural changes in tissue assembly and the SHG sensitivity to these collagen alterations, quantitative discrimination is achieved using small patient data sets. Ultimately these measurements may be developed as intrinsic biomarkers for use in clinical applications.

## Background

In 2008, there were an estimated 21,650 new cases of ovarian cancer in the United States and 15,520 deaths (Cancer Facts and Figures 2008, American Cancer Society, Database). Little is currently known about markers of premalignancy, early malignancy or early pathways for malignancy that could be potentially manipulated for prevention or early detection of ovarian cancer. Additionally, it is recognized that malignant cells with a specific and identifying molecular fingerprint are not always histologically identifiable in seemingly normal epithelium adjacent to tumor[1,2]. Moreover, certain subtypes of breast cancer are thought to carry the necessary gene expression characteristics that promote very early metastasis at the time that they become invasive[3], such that routine screening modalities will fail to detect them early enough to impact survival. This has also been suggested in high grade ovarian cancer with metastasis occurring much earlier than previously thought[4].

Thus there remains a compelling need for new technologies that have both sufficient resolution and specificity to detect microscopic tumors or precursor lesions. Probing alterations in the ECM composition and structure may be a promising approach in this regard, as these changes are thought to be critical for tumor initiation and progression for several epithelial carcinomas [5-7]. For example, up-regulation of several proteases (e.g. MMP2, MMP9, and uPa) in ovarian cancer have been implicated in invasion/metastasis where these act by degrading the basement membrane and/or stroma [8-13]. Additionally, in a feed-forward mechanism, changes in the stromal compartment of a tumor can then elicit a cascade of further changes involving fibroblasts and tumor cells thereby generating more aggressive tumor cells [14,15]. We propose that changes in the ECM may be a biomarker of invasion and provide insight into the factors that facilitate this process.

To investigate this possibility, we have explored the use of high resolution (~0.5 microns) Second Harmonic Generation (SHG) imaging microscopy[16] to objectively quantify differences in tissue structure in the ECM of normal and malignant ovarian tissues. SHG is a coherent nonlinear process wherein two lower energy photons are up-converted to exactly twice the incident frequency (or half the wavelength) of an excitation laser[17]. Like the more familiar two-photon excited fluorescence microscopy, this modality provides intrinsic optical sectioning and affords enhanced imaging depths into tissues (up to a few hundred microns)[18]. SHG does not utilize exogenous stains and, due to the underling physics of the contrast mechanism, directly visualizes the collagen assembly and is sensitive to changes therein[16,18-21]. The process results from a nonlinear polarization, rather than absorption, where this is given by:(1)

where P is the induced polarization, E is the electric field vector of the laser, and χ^(2) ^is the second order nonlinear susceptibility tensor of the collagen, and whose magnitude determines the contrast level. Due to the second order symmetry constraints imposed by Eq 1, the SHG contrast vanishes for assemblies with mirror symmetry (i.e. centro-symmetric environment) and increases for well-ordered structures[16]. Thus the relative alignment of fibrils/fibers is reflected in the magnitude of χ^(2) ^which is experimentally manifested in the SHG intensity. This tensor further contains information on the alignment of the collagen molecules in the fibrils/fibers[22]. Additionally, in contrast to fluorescence which is emitted at all angles, SHG has a well-defined emission direction that carries information related to the sub-resolution size and packing of the fibrils and fibers [23-27]. In this paper, SHG signatures of directionality, intensity, and polarization will be exploited to show differences in the structure of the ECM in normal and malignant human ovaries.

SHG has already been shown to have potential applicability for cancer diagnosis by revealing changes in the ECM in tumors relative to normal tissues. For example, the Dong[28] and Pavone[29] labs used SHG to identify tumor borders in basal cell carcinoma lesions by imaging the collagen assembly. Similarly, in a mouse model of breast carcinoma, Keely[30,31] identified distinct stages of invasion by measuring changes of the angle of collagen fibers with respect to tumor boundaries. Jain also demonstrated that the increased collagen concentration (i.e. desmoplasia) associated with a tumor from implanted melanoma cells was measurable by SHG [32]. As fibrillar type I collagen is by far the major structural element of the ovarian stroma, this modality may be well-suited for probing morphological and structural changes associated with early ovarian cancer. For example, Kirkpatrick used combined SHG and fluorescence imaging to show striking morphological changes in malignant human ovarian biopsies[33].

Here we take a more general approach of quantifying differences in the ECM of ex vivo normal and malignant human ovaries by using SHG to measure or extract changes in the respective fibril/fiber assemblies. The quantitative approaches to be shown in this paper avoid the pitfalls of empirical morphological assessments, and additionally avoid the problems with pure signal intensity based-measurements which can be confounded by scattering. Indeed, the magnitude and directionality of the scattering contains information on the tissue structure, which is encoded in the 3D SHG measurements[19]. Together, we define the SHG emission directionality (forward to backward ratio) and relative SHG intensity (conversion efficiency based on χ^(2)^) as the SHG creation attributes. These, together with the bulk optical parameters, are used in a comparative manner to quantify differences in tissue structure in normal and diseased ovary.

## Methods

### Tissue removal and preparation

The ovarian biopsies were removed using the University of Connecticut Health Center IRB approved protocol with patient consent prior to the procedure. The biopsies were removed and immediately fixed in 4% formalin and refrigerated for 24 hours. These specimens were then switched to PBS and kept at 4 degrees. For SHG imaging and bulk optical parameter measurements, un-stained sections of 100 and 50 microns, respectively, were cut with a vibratome. Independent classification of the ovary was based on histologic diagnosis of cancer (n = 3) or no known risk factors for normal ovaries (n = 5). All of the cancers were high grade and late stage (III or IV), although the tissue was harvested from the primary tumor in the ovary.

### Measurement of bulk optical parameters

The scattering coefficient μ_s_, and scattering anisotropy, g, are part of the metric to characterize the changes in tissue structure in normal and malignant ovaries. The inverse of μ_s _is the mean free path (MFP), and is the distance a photon will propagate before undergoing a scattering collision and changing direction. This anisotropy, g, is related to the directionality of the scattering, and varies from 0 to 1. The upper limit corresponds to highly forward-directed scattering and is reflective of very highly ordered tissues, and 0 represents the isotropic scattering associated with randomly organized structures. These bulk optical parameters (plus the absorption coefficient μ_a_) were determined at the 890 nm laser excitation wavelength using the titanium sapphire laser (see below) and the approximate SHG (457 nm Argon ion line) wavelengths using a dual integrating sphere approach and angular resolved scattering measurements as previously reported[19]. The refractive indices were determined using the method of Li[34]. These parameters were then used in an inverse Monte Carlo simulation[19,35,36] to calculate the absorption coefficient μ_a _and reduced scattering coefficient μ_s_' where:(2)

### SHG imaging system

The SHG imaging system has been described in detail elsewhere and is only briefly described here[22]. The instrument consists of a laser scanning unit (Fluoview 300;Olympus, Center Valley, PA) mounted on an upright microscope stand (BX61, Olympus), coupled to a mode-locked Titanium Sapphire femtosecond laser (Mira; Coherent, Santa Clara, CA). All SHG imaging was performed with an excitation wavelength of 890 nm with an average power of ~20 mW at the specimen using a water immersion 40× 0.8 NA objective. This excitation wavelength was chosen to provide good depth of penetration and also to exclude most of the potentially confounding sources of two-photon excited autofluorescence, which are predominantly excited at shorter wavelengths. This wavelength and NA resulted in lateral and axial resolutions of approximately 0.7 and 2.5 microns, respectively. Except for the polarization anisotropy measurements where linear polarization was used, SHG images were obtained using circularly polarized excitation as this probes all fiber orientations equally. The desired polarization at the focus was achieved as previously described[37]. The microscope simultaneously collects both the forward (F) and backward (B) components of the SHG intensity using identical calibrated detectors (7421 GaAsP photon counting modules; Hamamatsu, Hamamatsu City, Japan). The SHG wavelength (445 nm) was isolated with a 10 nm wide bandpass filter (Semrock, Rochester, NY). 3D renderings were performed with Imaris (Bitplane, Zurich Switzerland).

### 3D SHG imaging measurements

The measured depth dependence of the forward-backward intensity ratio (F/B) of the SHG signal is one means to characterize structural changes in the ECM between normal and malignant ovarian biopsies. This axial response arises from a convolution between the initial SHG directional emission ratio (which we denote F_SHG_/B_SHG_) and subsequent SHG propagation through the tissue which is based on μ_s _and g at 445 nm. The F_SHG_/B_SHG _is highly dependent upon the fibril diameter, the packing density and regularity relative to the size-scale of the SHG wavelength[23]. The bulk optical properties are related to density (primarily μ_s_) and organization (primarily g) of the fibrillar assembly. The measured SHG directional (F/B ratios) values were determined by integration of the intensity of 5-10 frames per optical section every few microns of depth using ImageJ software http://rsb.info.nih.gov/ij/.

The measured attenuation, i.e. rate of intensity decrease with increasing depth into tissue, of the forward SHG signal is also used to characterize structural changes in the ECM. The attenuation results from a compounded mechanism of SHG creation attributes (the F_SHG_/B_SHG _emission directionality and relative SHG intensity) and primary filter effect (loss of laser intensity due to scattering) and secondary filter effect (loss of SHG signal). Since biological tissues have intrinsic heterogeneity in concentration, we have found a normalized approach necessary to account for local variability in SHG intensities in the same tissue (different fields) and to make relative comparisons between tissues[19]. To this end the data of each optical series for each biopsy is self-normalized to the optical section with the average maximum intensity. The normalized forward attenuation data was taken concurrently with the F/B data. Like the directional data, these measurements are quantitated using ImageJ by integration over whole fields of view of the 40× lens.

### Monte Carlo Simulations

The 3D SHG directional and attenuation measurements are a convolution of the SHG creation attributes and the bulk optical parameters. These contributing factors are not directly determinable but can be decoupled by using Monte Carlo simulations. To this end, we have adapted the MCML framework[38] of photon propagation (absorption, scattering) to analyze the SHG axial responses[19,39]. First, the transmission of the laser, at a given focal depth, is determined based upon bulk optical parameters at 890 nm, and the cone formed by the NA of the lens. A relative SHG conversion efficiency, which is proportional to the second order nonlinear susceptibility χ^(2) ^values for the different tissues, determines the initial SHG intensity. An initial emission directionality, F_SHG_/B_SHG_, is also assumed. The propagation of the SHG signal (intensity and direction) is then calculated using the measured bulk optical parameters at the SHG wavelength. Collectively, by analyzing the trajectories of 50,000 photons at each focal depth, these steps simulate the detected directionality (F/B) and attenuation as a function of depth into the tissue.

## Results

### Assessment of morphological changes in the ECM by SHG imaging

**Figure 1:**
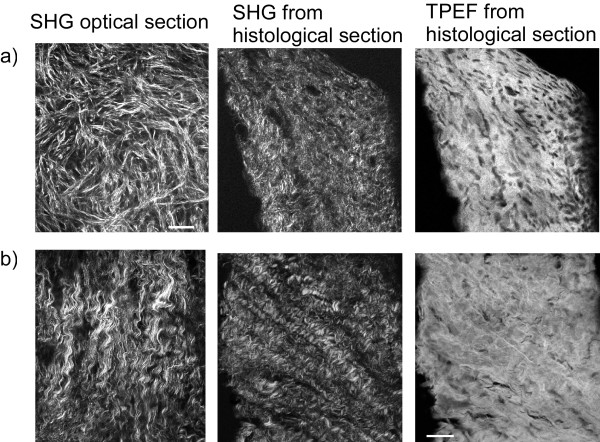
**Representative SHG and TPEF images from normal (a) and malignant (b) ovarian biopsies**. The left panels are *en face *SHG images of single optical sections where the tissue thickness was ~100 microns; the center and right images are SHG and TPEF images respectively from H&E optical sections from the same tissue as used for the *en face *images. Scale bar = 25 microns.

We first present qualitative evidence that SHG imaging has the sensitivity and specificity to reveal distinct collagen fibrillar assemblies in un-stained normal and malignant *ex vivo *biopsies. The left panels in Figure 1a and Figure 1b show respective SHG single optical sections from a normal ovary and a malignant tumor arising within an ovary. Large morphological differences in the collagen assembly are observed, where the fibers are "cross-hatched" in the normal, where in contrast they form highly regular helical structures in the tumor specimens. Images from different regions of the same normal ovary or the same tumor are characterized by similar respective morphology, and in addition, the morphologies displayed from tissues from different patients of each type are also similar.

To place these images in context, we compared them to standard H&E histopathology cross sections (5 microns thick) from the same respective biopsy. The middle and right panels of Figure 1 show representative results for forward detected SHG and two-photon excited fluorescence (TPEF) from these sections. The staining does not contribute to the SHG contrast, which arises from the collagen itself. The SHG images from the histological preparations do not appear the same as the left panel which showed *en face *optical sections from thicker tissue slices. Still, different fibrillar assemblies are observed in cross section as well. The TPEF channel primarily arises from eosin which stains essentially all proteins, which in the stroma is predominantly type I collagen. Thus for both the normal and cancer cases the TPEF from eosin and SHG reveal the same respective overall image features. However, the eosin contrast is less well-defined, which is likely a consequence of the sensitivity of SHG to fibril/fiber assembly, where, in contrast, there are no symmetry constraints on the fluorescence. This overlap gives us confidence that we can look at similar features by SHG as by histology, and, as we will show below, with enhanced information content. In addition, the ability to image intact thicker tissues in situ (100-200 microns) by SHG allows for the acquisition over more fields of view than is practical by conventional histology.

**Figure 2:**
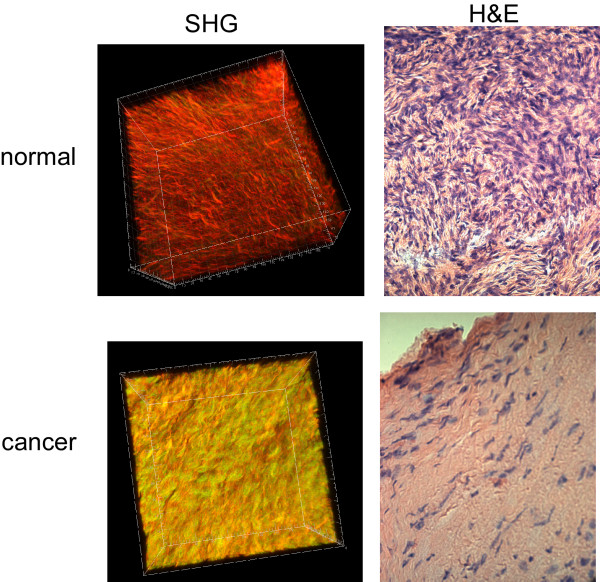
**3D SHG renderings (left panels) and H&E staining (right panels) of normal (top) and malignant ovarian biopsies (bottom)**. The field size for the 3D renderings was 170 microns and the histology cross sections were captured at 40×.

Next, we compare 3D renderings of SHG images with histological cross sections from the same biopsies (for both normal and malignant tissues) to examine the respective cell content and corresponding spatial distribution in the ECM (Figure 2). This is an important characterization as cells are transparent in SHG contrast, whereas the hematoxylin and eosin visualizes the cell nuclei and protein content respectively. Compared to the normal tissue (Figure 2, top), the cancer (Figure 2, bottom) is highly a-cellular away from the surface epithelium, consisting almost exclusively of dense collagen. This densely packed structure is also apparent in the 3D SHG rendering showing tightly packed helical fibrils (as also seen in the single optical section in Figure 1). By contrast, the normal tissue is less tightly woven, where the spaces accommodate stromal cells. We will show below that changes in collagen content between the tissues can be quantitatively probed, separately and in conjunction, through 3D SHG imaging and bulk optical parameter measurements.

### Bulk optical parameter measurements

The large difference in fiber morphology and collagen packing in malignant biopsies observed in both the SHG and H&E histology images suggests that the scattering coefficient and scattering anisotropy may be different than that of the normal tissue. The bulk optical parameters (μ_s_, μ_s_', μ_a_, and g) for the normal and malignant tissues at the laser (890 nm) and SHG (457 nm) wavelengths are given in Table 1 and the associated p values from two sided t-tests are shown in Table 2. For both tissues at both wavelengths, absorption (μ_a_) is negligible (~25-50 fold smaller) compared to scattering (μ_s_) and we therefore we have focused our attention on the latter. The malignant tissues are more highly scattering at the SHG wavelength compared to the normal (267 vs 172 cm^-1^) where this difference was significant (p = 0.008). We interpret the higher scattering of the cancer to be indicative that the matrix is more densely packed than the normal tissue, resulting in a smaller MFP. This conclusion is also borne out by inspection of the SHG renderings and H&E staining (Figure 2). We point out that these measurements were on fixed tissues, where the procedure will alter the absolute values of the scattering coefficient. However, previous EM studies have shown that fixation does not significantly alter the fibrillar structure and only results in a slight reduction in volume (~20%)[40]. We have also compared SHG images of fully hydrated and fixed specimens of tendon and skin, and found that the fibrillar morphology was similar with comparable associated shrinking (un-published data). We further note that in this analysis we are making comparisons between two tissues, where both of which would be similarly affected by the fixation procedure.

**Table 1:** Measured bulk optical parameters at the SHG and fundamental wavelengths with standard deviations.

	Cancer n = 3 457 nm	Cancer 890 nm	Normal n = 5 457 nm	Normal 890 nm
μ_s _(cm^-1^)	267 ± 19	195 ± 26	172 ± 39	161 ± 43

μ_a _(cm^-1^)	6.3 ± 3.1	6.6 ± 1.5	7.3 ± 2.4	5.6 ± 1.2

g	0.82 ± 0.02	0.86 ± 0.02	0.83 ± 0.01	0.94 ± 0.01

μ_s_' (cm^-1^)	46.2 ± 2.0	29 ± 3.3	29.6 ± 6.5	10.3 ± 3.8

**Table 2:** p values for the measured bulk optical parameters at the SHG and fundamental wavelengths

Wavelength	μ_s_	μ_a_	g	μ_s_'
890 nm	0.267	0.29	0.002	0.0004

457 nm	0.008	0.62	0.40	0.02

The scattering anisotropy values for the normal and malignant tissues at 890 nm and 457 nm and corresponding t-tests are shown in Tables 1 and 2, respectively. The g values are statistically different at the laser 890 nm wavelength (0.94 vs 0.86; p = 0.002) but are not different at the SHG wavelength (p = 0.4). We stress that the measured g values are to be considered effective, as the 50 micron thick biopsies can support ~1 scattering event (based on μ_s_), thus the actual values might be somewhat higher. However, as we have not extracted different values for ovarian tissues of 50 or 100 μm in thickness, we do not believe this to be a significant effect. This was also shown to be valid in prior work on dermis[19]. Moreover, the measurements are used for comparative analysis between the tissues, and as they were performed in a consistent manner, small absolute errors do not affect the following spectral data interpretation or subsequent Monte Carlo simulations.

While not all the respective μ_s _and g values for the normal and malignant tissues were different at the laser and SHG wavelengths, we first note that the redcued scattering coefficient μ_s_' was different at both wavelengths (890 nm, p = 0.0004; 457 nm, p = 0.02). Additionally, the spectral dependence or spectral slope[41] of μ_s_' was different where the malignant and normal tissues were characterized by a 1.5 and 3 fold respective increase in μ_s_' between the laser wavelength and SHG wavelengths. This flatter spectral slope for the more ordered malignant tissue (exemplified by highly periodic helical fibrils compared to the more randomly appearing normal tissue) is predicted by the recent theoretical treatment by Backman [42]. We have also seen this result in measurements on tendon, which has similar regularity[39]. Thus the spectral dependence of μ_s_' provides one piece of quantitative evidence of the change in tissue structure that occurs during ovarian cancer. These bulk optical parameters are also incorporated in Monte Carlo simulations of the depth dependent SHG directionality and attenuation shown below.

### SHG directional measurements

**Figure 3:**
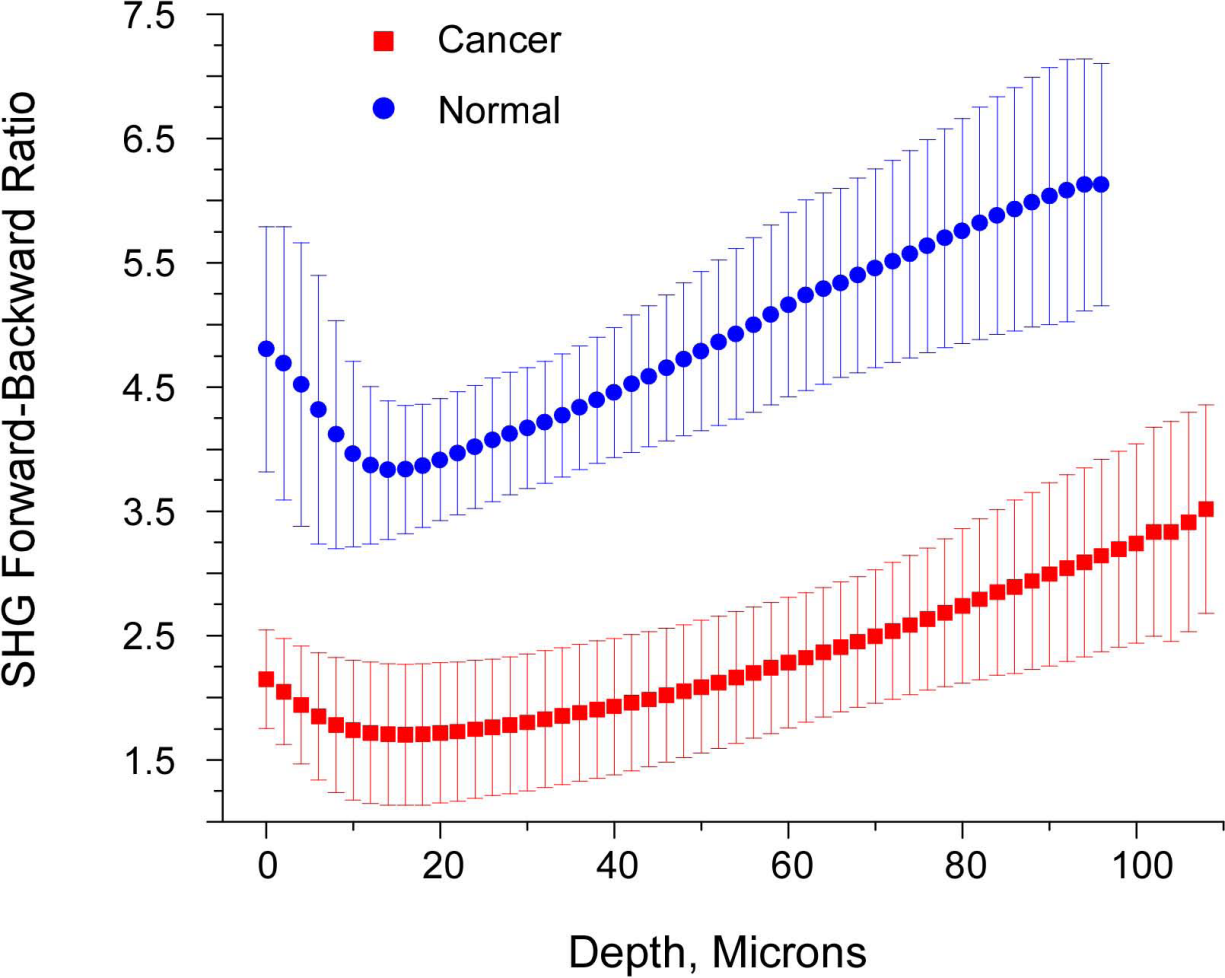
**Averaged measured forward/backward SHG intensities as a function of depth for normal (blue circles) and malignant (red squares) ovaries**.

The measured forward-backward intensity ratio (F/B) arises from a convolution between the SHG directional emission creation ratio (F_SHG_/B_SHG_) and subsequent signal propagation through the tissue based on μ_s _and g at the SHG wavelength. The averaged experimentally measured F/B vs depth plots for normal (n = 5) and malignant ovaries (n = 3) are shown in Figure 3. At all depths below the surface epithelium the SHG from the normal tissues are more forward directed than the cancers, which is consistent with the lower scattering coefficient of the former (172 vs 267 cm^-1^), such that photons that are initially forward directed have a higher probability of continuing to propagate in this direction. To validate the distinction between these tissues t-tests were performed at 10 micron depth intervals, and the differences were statistically significant (p < 0.01 in all cases).

We also observe that for both tissues the F/B increases with increasing depth into the tissue. This result is consistent in the framework of photon diffusion theory, where at least one transport MFP is required between the location of the emitted photon and the forward boundary of the specimen for efficient multiple scattering to occur[38]. Thus as the distance from the focal point to the tissue boundary shortens, the probability of multiple scattering events decreases and the F/B ratio subsequently increases.

**Figure 4:**
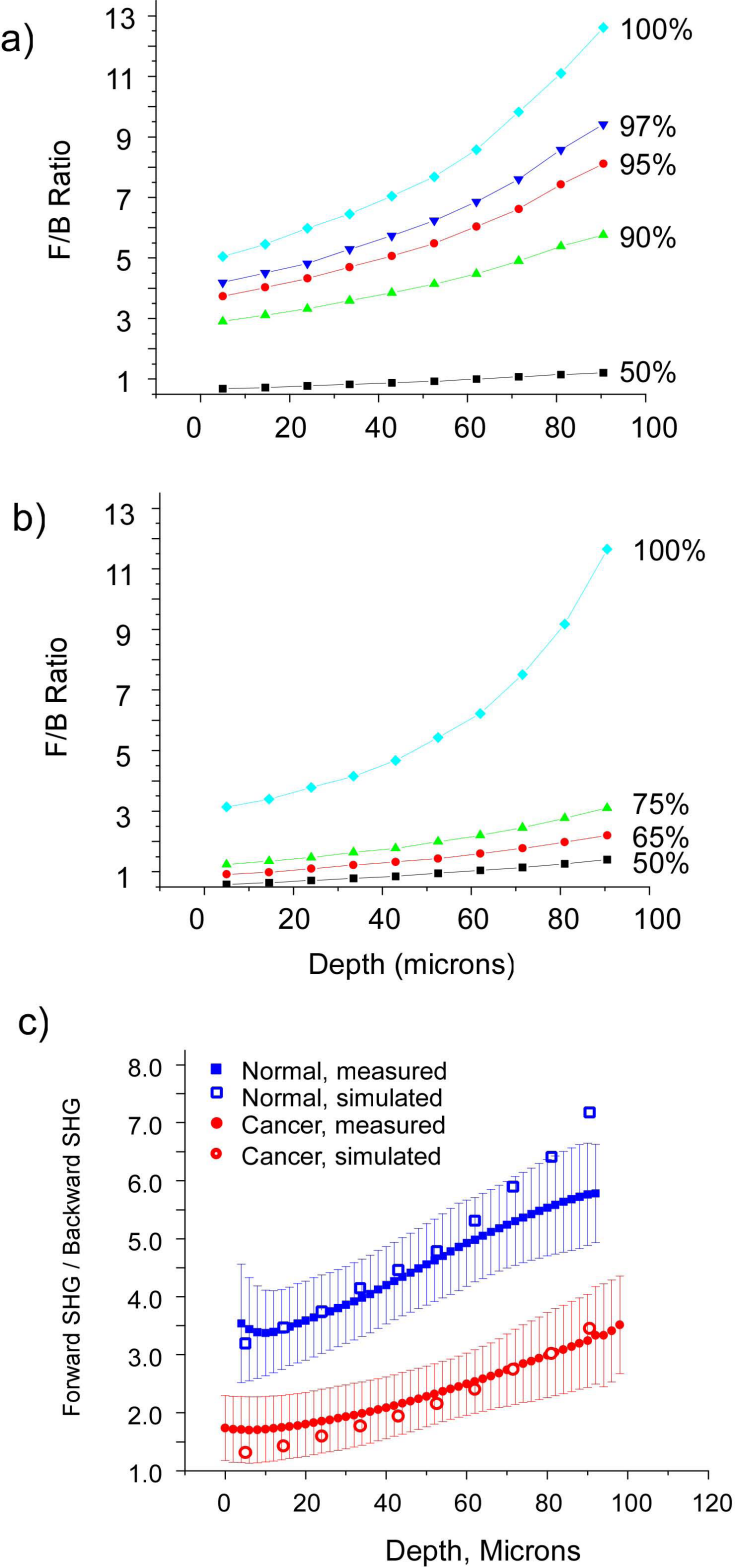
**Monte Carlo Simulations of the measured F/B response, where (a) and (b) show the results for normal (a) and malignant (b) ovaries using the bulk optical parameters in Table 1 over a range of initial emission distributions**. The best fit simulation to the data in each case is overlapped with the experimental data in (c), where the %F_SHG _was determined to be 77% and 93% for the malignant and normal tissues, respectively.

We utilized Monte Carlo simulations of these plots using the measured bulk optical parameters at inputs to decouple the initial emission directionality (i.e. F_SHG_/B_SHG_) from the SHG propagation (based on μ_s _and g). To estimate this creation attribute we ran simulations varying the forward emitted fraction (F_SHG_) from 50-100%. Representative simulations for the normal and malignant biopsies are shown in Figure 4a and 4b, respectively, which demonstrate the pronounced effect the initial directionality has on the measured F/B. We then fit to the initial directionality by squaring and summing the residuals between the series of simulations and the experimental data. Taking the minimum of the R^2 ^function yielded %F_SHG _of 93% and 77%, for the normal and cancer, respectively, where the uncertainly in each case is approximately ± 3%. The corresponding Monte Carlo simulation generated from the best fit for each tissue type (open squares = normal and open circles = cancer) are overlapped with the experimental SHG data in Figure 4c. The Chi-squared test between the experimental and simulated data resulted in values of 0.40 and 0.30 for the normal and cancer respectively, indicating that, for both tissues at the α = 0.05 level, the differences between the data and corresponding simulation are not significantly different. This good fit between the simulated and measured data thus gives us confidence in the extracted %F_SHG _values.

The SHG emission directionalities between the tissues are significantly different and can be interpreted by the difference in fibril assembly. Using a space filling analysis of TEM images we found that the packing of the fibrils in the malignant tissue to be more regular relative to the normal (10% vs 15% inter-fibrillar space respectively). Based on our mathematical model of SHG in fibrillar tissues,[23] the fibril assembly of the malignant tissue, i.e. regularly packed fibrils on the order of the coherence length, would give rise to efficient backward emitted SHG[23]. In contrast the more random assembly in the normal would result in more predominantly forward initial emission directionality (i.e. higher %F_SHG_), as was extracted from the simulation of the data.

### SHG attenuation measurements

**Figure 5:**
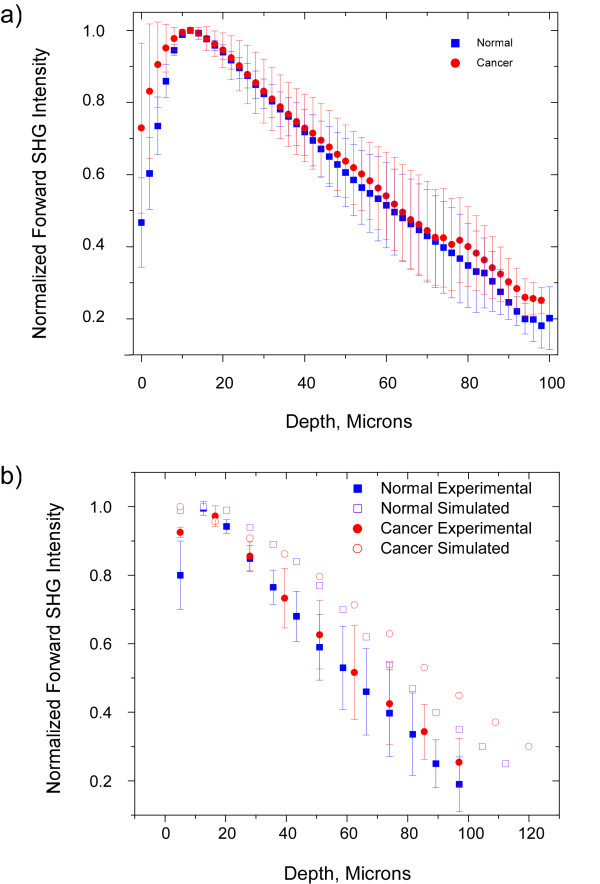
**Forward SHG attenuation data and simulations for normal and malignant ovarian biopsies**. 5(a) shows the experimental data for normal (blue squares) and cancer (red circles). 5(b) shows the experimental data (closed circles and squares) every 10 microns and the simulations (open circles and squares) based on the measured bulk optical parameters in Table I and the relative χ^(2) ^values that were determined from the histological sections.

The averaged normalized forward attenuation data with standard errors are shown in Figure 5a for the normal (n = 5) and malignant (n = 3) tissues. Unlike the F/B response, the SHG attenuation provides no clear separation between the tissues. To understand this effect, we need to consider all the factors that give rise to the measured attenuation. This results from a compounded mechanism of the F_SHG_/B_SHG _emission directionality, the relative SHG intensities governed by the relative magnitudes of the χ^(2) ^nonlinear susceptibility tensors, and primary and secondary filter effects from the respective bulk optical properties. As we have previously reported,[19] it is not possible to directly determine relative χ^(2) ^values in intact tissues as the measured signal is convolved with scattering when the tissues are thicker than one MFP or ~50 microns. However this can be achieved using much thinner histological sections (~5 microns). We note that the eosin staining does not contribute to the observed SHG. Measurement of the relative SHG intensities from these sections yields a factor of 3.9 ± 0.1 (p < 0.005) increased brightness for the cancer over that of normal tissue.

We now use all our measured factors as inputs into Monte Carlo simulations of the SHG attenuation. The simulated data for the normal (open squares) and malignant tumors (open circles) based on the bulk optical parameters (see Table 1) and the relative χ^(2) ^values are shown in Figure 5b. Like the experimental data, the simulations are highly similar for these two tissues. However, the rate of decay of the simulated SHG intensity is somewhat greater for the experimental data, where the differences are most pronounced at the bottom of the slice. While an exact match was not obtained this approach still allows us to understand the similarity in the measured data for these tissues. This similarity arises from the offsetting parameters of the increased conversion efficiency (χ^(2)^) and larger μ_s _for the cancers, as these separately would result in slower and faster normalized attenuations, respectively. We point out if the relative SHG conversion efficiencies were not independently known, this approach would allow this determination once the respective bulk optical parameters were measured at the laser excitation and SHG wavelengths. This would be accomplished by running simulations varying the relative conversion efficiency and then comparing the results to the experimental data to achieve the best fit (in analogy with the method presented on the directional data).

## Discussion

### SHG imaging to measure changes in tissue structure

Our goal in this work was to develop SHG imaging and analysis metrics that can be used to understand changes in ECM structure during carcinogenesis and eventually be utilized in clinical applications. The enabling aspects of SHG for tissue characterization lie not solely in the ability to visualize the fibrillar morphology, but also in the contrast being reflective of the supramolecular and fibrillar structure of collagen [16,18]. These differences in structural features cannot be resolved by optical microscopy methods but they are manifested in terms of the relative SHG intensities (i.e. χ^(2) ^values) and initial emission directionality (F_SHG_/B_SHG_). For example, these creation attributes depend on packing density and order of the inter-fibril structure, both of which change in cancer (Figure 1). Monte Carlo simulation of the 3D SHG responses incorporating the bulk optical parameters allows the isolation of these attributes and provides insight into the tissue assembly. For example, the directional measurements are most sensitive to F_SHG_/B_SHG _which is related to the fibril size and packing, and was different for both tissues. By contrast, the attenuation measurements are not appreciably affected by this term but are highly sensitive to the relative χ^(2) ^values, which are related to collagen concentration and assembly, which was also different for both tissues. The directly measured or extracted SHG attributes or bulk optical properties that are different for the normal and malignant ovaries along with the physical interpretation are summarized in Table 3. In contrast, in our prior work we showed that when Osteogenesis imperfecta (a connective tissue disorder arising from abnormal collagen) skin was compared to the normal, a different set of parameters was found to differentiate them[19]. Thus the depth dependent directionality (F/B), the attenuation measurements, bulk optical parameters, and corresponding simulations represent a versatile means for characterizing changes in tissue structure occurring in diseases in which collagen alterations occur.

**Table 3:** Summary of structural properties different in normal and malignant ovaries

	μ_s _(457 nm) (cm^-1^) density	Refractive index correlation function	SHG emission (%forward) Fibril packing Into fibers	SHG Intensity (relative) Concentration and organization	SHG anisotropy Fiber organization
Normal	172 ± 39	Mass fractal	93%	1	0.76

Cancer	267 ± 19	Stretched exponential	77%	3.9	0.88

Conclusion	Cancer denser	Cancer more ordered	Cancer better packed fibrils	Cancer denser and more organized	Cancer more organized

While the attenuation measurement did not provide clear discrimination between the normal and malignant tissues, we can better understand the measured data through the use of simulations. We note that in the cancer biopsies, the tumor extensively replaced the ovary (near the surface) and it is possible there could be discrimination in earlier stage disease, where both the SHG and bulk optical parameters could likely be different due to different levels of ECM remodeling. Specifically, we predict that earlier stage tumors would be characterized by decreased scattering and SHG brightness relative to the latter stage tumors imaged here. Moreover, even for these tissues, the attenuation response at other wavelengths may provide discrimination due to the differences in the spectral slope of μ_s_' (see Table 1 of bulk optical properties).

### Collagen remodeling in ovarian cancer

Collectively, the SHG creation attributes, bulk optical parameters, and cell density (by H&E histology) suggest that a profound alteration of the ECM occurs in ovarian cancer. An unanswered and important question is if this structure represents remodeled existing collagen or new synthesis by the stromal cells, or both. There is significant translational importance to this question as it addresses the temporal evolution of invasion. To begin to address this question, we first note that the SHG intensity was nearly 4 fold greater in the malignant ovary, suggesting the collagen was both higher in abundance and more organized. We have investigated the fibril size and packing using TEM and found similar size distributions of fibril diameter in the normal and malignant biopsies, where the most probable diameter was ~80 nm. However, the fibrils in the tumor are more closely packed, which would result in a greater SHG intensity.

**Figure 6:**
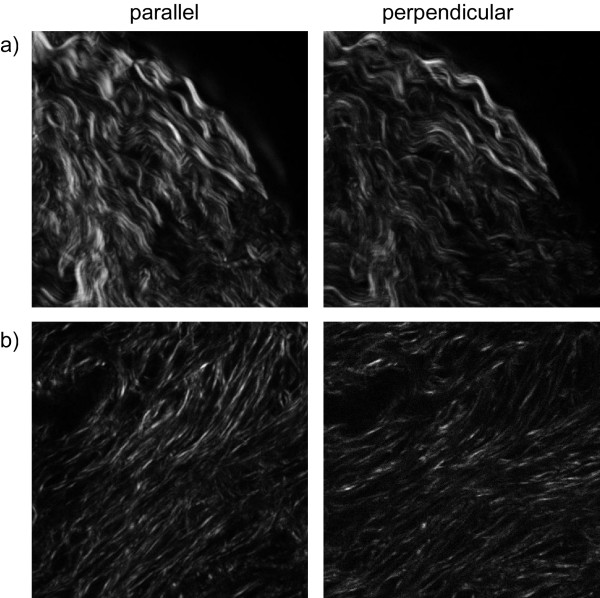
**SHG anisotropy measurements of cancer (a) and normal (b) ovary, where the left and right panels are images acquired with a polarizer parallel and perpendicular to the laser polarization, respectively**. Field size = 170 microns.

We can next assess the regularity of the collagen fiber alignment by measuring the SHG anisotropy. This anisotropy, β, is calculated by:(3)

where I_par _and I_perp _correspond to the SHG intensity detected after a polarizer oriented parallel and perpendicularly to the laser polarization, respectively. Here the linear polarization of the laser is fixed at 45 degrees relative to the fiber axis (on a fiber by fiber basis) and then the SHG parallel and perpendicular components are measured relative to this excitation polarization. We chose this angle because this orientation yields the strongest SHG intensity for collagen[22]. Values of β vary from -0.5 to 1 where 0 represents completely random organization and 1 represents completely ordered fibers. We have acquired polarization analyzed data from forward SHG and calculated β values of 0.88 and 0.76 for the malignant and normal ovary, respectively, where representative analyzed optical sections are given in Figure 6. The higher β for the cancer corresponds to greater fiber alignment, where this assembly would also yield brighter SHG, as was observed through imaging of the thin sections.

While this issue warrants much further investigation, the existing data based on similar fibril diameter (based on TEM) and increased fibril/fiber organization supports the argument that the collagen beneath the surface epithelium is more likely to be newly synthesized as opposed to existing collagen fibrils being remodeled by proteases. This is also indicated by the spectral dependence of μ_s_' between the laser excitation and SHG wavelengths. Using the formalism of Backman,[42] we find that the normal and malignant tissues are characterized by different refractive index correlation functions. The malignant tissue is characterized by a stretched exponential function, where the scattering objects are similar in size[42]. The normal tissue falls within the mass fractal regime, which is best described as having only characteristic length scales over which there is similarity in the refractive index, rather than typical scattering sizes. In sum, based on the separate scattering and SHG measurements, it would appear unlikely that less ordered collagen in normal stroma could be transformed into a more ordered structure in a tumor.

### Comparison to fluorescence microscopy

We have achieved statistical discrimination in the measured F/B directional data based on a small data set of normal and malignant ovaries because the optical signatures are profoundly different in these tissues but homogeneous within each type. Moreover, the SHG creation attributes and bulk optical properties are consistent with the differences in fibrillar assembly between the tissues based on the morphology observed by H&E, and EM. By contrast, inherent variability and non-specificity in modalities such as fluorescence precludes this definitive distinction. For example, a previous paper on ovarian cancer found significant heterogeneity between diagnostic groups arising from innate tissue variations[43]. The authors concluded that fluorescence measurements at several different wavelengths were required for characterization, and that larger numbers of patients were needed to develop this modality as a robust imaging tool based. In fact, because of this heterogeneity, fluorescence imaging (especially of metabolism) is now more commonly being combined with other modalities to improve the specificity of optical measurements for many epithelial cancers including ovarian[33]. We did not find this degree of variability present in our small study, which implies that the specific signal that SHG measures has far less heterogeneity than would be expected from fluorescence data alone. This may arise as SHG only probes the collagen assembly, whereas the autofluorescence can arise from many components. We note that while collagen autofluorescence has been detected spectroscopically from tissues, is typically too weak to be useful for microscopic imaging.

### Outlook

Through this exploratory study derived from a basic science perspective we have identified a collection of physical/structural properties of the ECM that change when the ovary becomes malignant. This characterization was enabled by the high sensitivity/specificity of SHG to collagen assembly. Our longer term goals are to use these structural changes in the ECM as a specific biomarker in vivo. While at the 890 nm excitation wavelength there was no discrimination between normal and diseased ovary in the forward attenuation data, this may be different at other wavelengths due to the spectral slope differences (i.e. from the different refractive index correlation functions) as well as different stages of disease. Moreover, performing these measurements in the backward collection geometry would yield all the same data as shown here in the forward case. With our current technology, the directional measurements cannot be performed in vivo, however given the rapid advances in fiber-optics and miniaturized scanning devices we envision that a multiple probe scheme could be constructed where the same information could be extracted. We note that the directional measurements could be applied to ex vivo biopsies of more accessible epithelial tissues such as skin and breast, where ECM remodeling also accompanies carcinogenesis.

## Conclusions

We have performed an exploratory study to understand and quantify changes in tissue structure that occur during ovarian carcinogenesis. In this effort we have identified optical signatures (SHG creation attributes and bulk optical parameters) that are different between normal and malignant ovarian tissues and have established their relative sensitivities. We found the depth-dependent directional and attenuation responses are consistent with the differences in tissue structure at both the fibril and fiber levels of assembly. The increased order of the fibril/fiber assembly suggests that in the malignant tissues that the collagen has been newly synthesized. We find using surprisingly small patient data sets that our methods provide statistical discrimination between normal and malignant ovarian biopsies in terms of the SHG creation attributes as well the bulk optical parameters, pointing to the specificity of the approach. We anticipate that the methods will prove valuable in terms of better investigating changes in the ECM structure which will impact our understanding of early disease. We lastly suggest that SHG imaging may become a powerful tool for future diagnostic applications as an early biomarker for the ovary and potentially other epithelial cancers.

## Abbreviations

ECM: extracellular matrix; EM: electron microscopy; F/B: measured forward to backward ratio; F_SHG_/B_SHG_: emitted forward to backward ratio; g: scattering anisotropy; MCML: Monte Carlo multilayer; MFP: mean free path; NA: numerical aperture; SHG: Second Harmonic Generation; H&E: hematoxylin and eosin; TEM: transmission electron microscopy; TPEF: two-photon excited fluorescence; μ_s_': reduced scattering coefficient; μ_s_: scattering coefficient.

## Competing interests

The authors declare that they have no competing interests.

## Authors' contributions

ON prepared the specimens, acquired, process and analyzed the spectroscopic and image data, RBL performed the Monte Carlo simulations, MAB co-conceived the project, acquired the tissue specimens, and helped draft the manuscript, PJC co-conceived the project and primarily drafted the manuscript. All authors read and approved the final manuscript.

## Pre-publication history

The pre-publication history for this paper can be accessed here:

http://www.biomedcentral.com/1471-2407/10/94/prepub
